# From mother to infant: predicting infant temperament using maternal mental health measures and tabular machine learning models

**DOI:** 10.3389/fpubh.2025.1659987

**Published:** 2025-09-18

**Authors:** Rawan AlSaad, Majid Alabdulla, Aliya Tabassum, Javaid Sheikh, Rajat Thomas

**Affiliations:** ^1^Weill Cornell Medicine-Qatar, Doha, Qatar; ^2^Mental Health Services, Hamad Medical Corporation, Doha, Qatar; ^3^College of Medicine, Qatar University, Doha, Qatar; ^4^College of Engineering, Qatar University, Doha, Qatar

**Keywords:** artificial intelligence, machine learning, maternal mental health, infant temperament, postpartum depression, women's health, depression, anxiety

## Abstract

**Background:**

Negative emotionality is a core dimension of infant temperament, characterized by heightened distress, reactivity, and difficulty with self-regulation. It has been consistently associated with later behavioral and emotional difficulties. Emerging evidence suggests that maternal mental health (MMH) in the postpartum period may influence infant temperament. However, few studies have applied machine learning (ML) methods to examine the predictive capacity of MMH profiles for early infant emotional development.

**Objectives:**

This study aimed to investigate whether postpartum maternal depression, anxiety, and birth-related trauma, along with sociodemographic factors, can predict infant negative emotionality during the first year postpartum using tabular ML models.

**Methods:**

Data were obtained from 410 mother–infant dyads. Infant temperament was assessed using the Negative Emotionality subscale of the Infant Behavior Questionnaire-Revised (IBQ-R). MMH symptoms were measured via the Edinburgh Postnatal Depression Scale (EPDS), the Hospital Anxiety and Depression Scale (HADS), and the City Birth Trauma Scale (City BiTS). Six tabular ML models were trained using MMH and demographic features: Tabular Prior-Data Fitted Network (TabPFN), Light Gradient Boosting Machine (LightGBM), eXtreme Gradient Boosting (XGBoost), Categorical Boosting (CatBoost), Random Forest, and Support Vector Machine (SVM). Performance was evaluated using Receiver Operating Characteristic Area Under The Curve (ROC-AUC), Precision-Recall Area Under the Curve (PR-AUC), F1-score, sensitivity, and specificity.

**Results:**

Postpartum MMH symptoms and maternal–infant characteristics moderately predicted infant negative emotionality. LightGBM achieved the highest performance across ROC-AUC (0.76), F1-score (0.72), sensitivity (0.71), and specificity (0.73). TabPFN yielded the highest PR-AUC (0.78). Key predictors included gestational age, infant's age, EPDS score, mother's age, HADS score, and City BiTS score.

**Conclusions:**

These findings highlight the potential of ML tools in early identification of at-risk infants and the importance of integrating MMH screening into postnatal care. Such predictive insights can inform timely, personalized interventions that address the unique emotional needs of both mother and infant, ultimately fostering healthier developmental trajectories and enhancing overall family well being.

## 1 Introduction

Infant temperament refers to early-appearing individual differences in emotional reactivity and self-regulation, observable within the first 12 months of life (1). Among the major dimensions of temperament, negative emotionality (often labeled “difficult” temperament) is characterized by heightened sensitivity to stress, frequent expressions of distress, irritability, frustration, and fearfulness (1). Importantly, these early-emerging patterns are not transient and research has shown that infant temperament, especially high negative emotionality, can have a lasting impact on developmental trajectories (2). Specifically, it has been associated with poorer emotional regulation skills, lower cognitive and academic performance, increased risk for behavioral problems, impaired peer relationships, and elevated vulnerability to internalizing and externalizing psychopathologies across childhood and adolescence (3, 4). Early identification of negative emotionality is, therefore critical, as it provides an opportunity for timely, targeted interventions that support both maternal well being and optimal infant developmental outcomes.

Maternal mental health (MMH) has long been linked with infant temperament development. Postpartum depression, in particular, shows a consistent association with perceived difficult infant temperament (5). Longitudinal research suggests this mother-infant dynamic can become bidirectional, whereby maternal depression contributes to infant fussiness, which in turn can exacerbate the mother's depression in a cyclical pattern (6, 7). Beyond depression, maternal anxiety (8) and stress (9) have also been implicated in infant temperament and are associated with poorer infant socio-emotional development.

Despite growing recognition of the impact of MMH on early child development, there remains a notable gap in the application of machine learning (ML) techniques specifically aimed at predicting infant temperament outcomes. Most existing ML studies in the perinatal domain have focused on broader infant health indicators rather than temperament dimensions. Nevertheless, ML offers substantial advantages over traditional statistical methods, particularly in its ability to model complex, non-linear interactions among psychological, demographic, and medical predictors. For example, Yang et al. (10) developed a combined model using random forests and multilayer perceptrons to examine whether maternal health indicators (including psychological well being) could predict infant behavioral characteristics and sleep quality. In addition, Punamaki et al. (11) examined how prenatal and perinatal mental health and medical conditions predict infant's developmental and health status at 12 months.

However, several key gaps exist in the current literature. First, few studies have framed the problem as a classification task, such as distinguishing infants at high vs. low risk for difficult temperament, as opposed to traditional correlational or regression-based analyses of continuous temperament scores. Second, there has been an underuse of modern tabular ML models, algorithms designed to handle structured clinical datasets, for predicting infant temperament outcomes. Third, there is a lack of studies that integrate comprehensive MMH profiles, including concurrent measures of depression, anxiety, and birth-related trauma, in conjunction with relevant maternal variables such as gestational age at birth and maternal age. Addressing these gaps by leveraging efficient tabular ML classification techniques and incorporating a more holistic array of maternal mental health risk factors is essential to enhance the early identification of infants at elevated risk for developing difficult temperament profiles.

**Figure 1:**
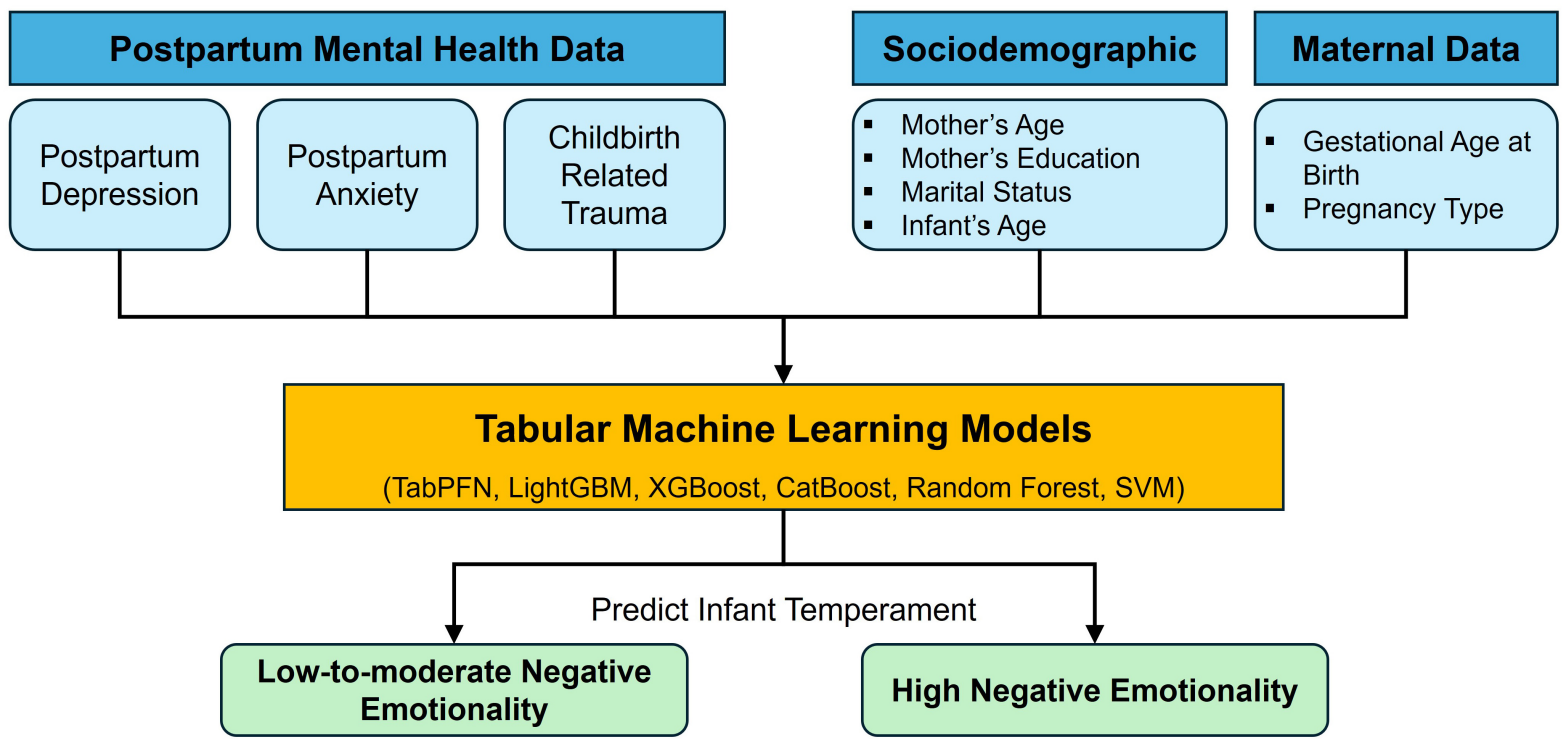
Overview of the study design for predicting infant negative emotionality based on postpartum mental health profiles, sociodemographic characteristics, and maternal data.

The present study investigates whether postpartum maternal depression, anxiety, and birth-related trauma can collectively predict infant negative emotionality during the first year postpartum using tabular ML models. We specifically ask: to what extent can MMH measures predict an infant's high negative emotionality in the first year? To answer this question, we analyzed data from 410 mother–infant dyads, applying six different tabular ML algorithms to classify infants into either “high” or “low-to-moderate” negative emotionality groups based on a standard temperament assessment (Figure 1).

## 2 Methods

### 2.1 Study population and data sources

This study utilized data from an open-access dataset (12) comprising 410 mother–infant dyads. Data were collected via a cross-sectional online survey conducted between June and September 2020 at a university hospital in Switzerland. Eligible participants were biological mothers aged 18 years or older with an infant aged 3–12 months at the time of participation and no history of major neonatal health complications. For this analysis, we used a subset of the dataset consisting of 60 variables, including six demographic and maternal characteristics, 10 items from the Infant Behavior Questionnaire-Revised (Negative Emotionality dimension), 10 items from the Edinburgh Postnatal Depression Scale, seven items from the Hospital Anxiety and Depression Scale, and 20 items from the Maternal City Birth Trauma Scale. A comprehensive description of the input features is provided in Appendix A.

### 2.2 Data elements

#### 2.2.1 Infant temperament measures

Infant temperament was assessed using the Negative Emotionality subscale (IBQ-NEG) of the Very Short Form of the Infant Behavior Questionnaire–Revised (IBQ-R VSF) (13). This validated maternal-report instrument captures caregivers' perceptions of specific, observable infant behaviors over the past weeks. The negative emotionality dimension captures the infant's tendency to express distress, sadness, fear, and frustration in response to limitations or unfamiliar situations. It comprises 12 items, with 10 of them included in our analysis as outlined in Appendix A.5. The IBQ-NEG subscale uses a 7-point Likert scale based on frequency of behavior, where 1 = never, 2 = very rarely, 3 = less than half the time, 4 = about half the time, 5 = more than half the time, 6 = almost always, and 7 = always.

Composite scores for negative emotionality were calculated by averaging responses to the 10 selected items from the IBQ-NEG subscale for each infant. To enable machine learning classification, these continuous scores were transformed into two categories based on a threshold of 3.4. This threshold was chosen based on the median score (50th percentile) of the sample distribution, a common and statistically grounded practice when stratifying continuous temperament measures in the absence of established clinical cutoffs. This approach enables a clear separation between higher and lower emotional reactivity, helping identify infants with greater distress tendencies. Although the IBQ-R does not specify clinical cut points, median splits have been widely used in prior studies to model high vs. low negative emotionality groups (14, 15). Infants scoring 3.4 or below were classified as Low-to-Moderate Negative Emotionality (Class 0; *n* = 202), typically displaying calm or moderately reactive behavior. These infants may show occasional fussiness or clinginess when tired or exposed to unfamiliar stimuli but generally recover well with caregiver support and demonstrate stable emotional regulation. Infants scoring above 3.4 were classified as High Negative Emotionality (Class 1; *n* = 208), characterized by frequent crying, heightened reactivity, and difficulty calming down—traits that may signal greater sensitivity to environmental stressors and a need for increased emotional support and structured caregiving.

#### 2.2.2 Maternal mental health measures

Data on maternal mental health were collected through a structured questionnaire, which also included basic demographic information such as the mother's age and education level. The assessment targeted three core domains: postpartum depressive symptoms, anxiety, and trauma related to birth. To ensure comprehensive evaluation, three well-established self-report instruments were used: the Edinburgh Postnatal Depression Scale (EPDS) (16), the Hospital Anxiety and Depression Scale: Anxiety subscale (HADS-A) (17), and the City Birth Trauma Scale (City BiTS) (18). These three tools collectively offered a multidimensional evaluation of maternal mental health following childbirth.

### 2.3 Data preprocessing

Preprocessing steps included calculating total scores for the EPDS, HADS-A, and City BiTS scales, imputing missing values, and recoding variables for consistency. Numerical features were mean-imputed and standardized, while categorical features were mode-imputed and one-hot encoded using a column transformer pipeline. This ensured the data were clean, complete, and ready for machine learning analysis.

### 2.4 Tabular machine learning models

To examine the predictive utility of postpartum maternal mental health (MMH) symptom profiles combined with sociodemographic variables, we utilized six tabular machine learning models: Tabular Prior-Data Fitted Network (TabPFN), Light Gradient Boosting Machine (LightGBM), eXtreme Gradient Boosting (XGBoost), Categorical Boosting (CatBoost), Random Forest, and Support Vector Machine (SVM). These models were selected to compare the performance of both classical and state-of-the-art tabular algorithms in classifying infant negative emotionality levels.

TabPFN (19) is a recent transformer-based deep learning model trained offline on millions of synthetic tabular tasks. It learns to make predictions using Bayesian model averaging, essentially simulating what an ensemble of classical models might output with a single forward pass. LightGBM (20) is a gradient boosting framework that improves speed and accuracy using histogram-based binning and leaf-wise tree growth strategies. XGBoost (21) is a highly efficient gradient boosting algorithm that builds decision trees sequentially, minimizing the residual errors of prior trees. It is known for its performance and regularization capabilities. CatBoost (22) is a gradient boosting algorithm that is particularly optimized for categorical features, making it well-suited for datasets with mixed feature types. It uses ordered boosting and target statistics to reduce overfitting and improve generalization. Random Forest is an ensemble method that constructs multiple decision trees on random subsets of the data and aggregates their outputs to produce robust predictions. SVM is a kernel-based algorithm that seeks the optimal hyperplane to separate classes in a transformed feature space. We implemented an SVM classifier using the radial basis function (RBF) kernel. The RBF kernel was chosen due to its ability to model non-linear relationships between input features and the outcome.

We selected gradient-boosted trees (LightGBM, XGBoost, and CatBoost), Random Forest, SVM, and TabPFN because the feature set mixes ordinal Likert items and categorical variables and likely exhibits non-linear effects and higher-order interactions. These models natively capture such structure without extensive manual feature engineering and typically perform strongly on medium-sized tabular datasets. We acknowledge that more inherently interpretable families can provide coefficient- or curve-level explanations, albeit with reduced flexibility for complex interactions. Given our screening objective, we prioritized predictive accuracy and addressed interpretability *post-hoc* via model-agnostic feature importance.

### 2.5 Evaluation setup

Model performance was evaluated using five key metrics: Area Under the Receiver Operating Characteristic Curve (ROC-AUC), Area Under the Precision-Recall Curve (PR-AUC), F1-score, sensitivity, and specificity. ROC-AUC was selected to measure the models' overall ability to discriminate between high and low-to-moderate negative emotionality across all thresholds. PR-AUC was included to highlight the balance between precision and recall, which is particularly important when correctly identifying at-risk infants is prioritized. F1-score, sensitivity, and specificity were reported to further capture performance trade-offs relevant for real-world screening applications, where both false positives and false negatives carry implications for care. Since the two outcome classes were nearly balanced (208 high vs. 202 low-to-moderate negative emotionality), we did not apply class weighting or any sampling techniques during model training. The dataset was partitioned into a training set (80%) and a testing set (20%) to assess generalizability. We employed repeated five-fold cross-validation with three repetitions, reporting the mean and standard deviation of each evaluation metric across the 15 validation folds to quantify performance variability.

## 3 Results

### 3.1 Participant characteristics

A total of 410 mother–infant dyads were included in the study. Participant characteristics and summary measures are presented in Table 1. The mean maternal age was 30.20 years (SD = 4.36). In terms of educational attainment, nearly half of the mothers (46.8%) held a university degree. The majority of participants were in a couple relationship (94.9%). The sample was nearly evenly split by infant gender, with 52% female and 48% male infants. The mean gestational age at birth was 39.1 weeks (SD = 1.90). At the time of assessment, infants were fairly evenly distributed across the three age groups: 3– < 6 months, 6– < 9 months, and 9– < 12 months. Regarding maternal mental health, the mean scores were 9.05 (SD = 6.76) on the EPDS, 7.84 (SD = 4.26) on the HADS-A, and 13.12 (SD = 10.81) on the City BiTS.

**Table 1:** Sample characteristics and key measures (N = 410).

**Variable**	**Value**
Maternal age (years)	*M* = 30.20, SD = 4.36
Gestational age at birth (weeks)	*M* = 39.11, SD = 1.90
**Marital status**	
Couple relationship	389 (94.9%)
Single	14 (3.4%)
Separated/Divorced/Widowed	7 (1.7%)
**Educational level**	
University degree	192 (46.8%)
Applied science/Tech diploma	88 (21.5%)
Post-secondary/apprenticeship	103 (25.1%)
Completed compulsory school	25 (6.1%)
No formal education	2 (0.5%)
**Infant gender**	
Female	212 (51.7%)
Male	198 (48.3%)
**Infant age group**	
3– < 6 months	147 (35.9%)
6– < 9 months	133 (32.4%)
9– < 12 months	130 (31.7%)
EPDS total score	*M* = 9.05, SD = 6.76
HADS-A total score	*M* = 7.84, SD = 4.26
City BiTS total score	*M* = 13.12, SD = 10.81
IBQ-R negative emotionality score	*M* = 3.36, SD = 1.10

### 3.2 Models performance

**Figure 2:**
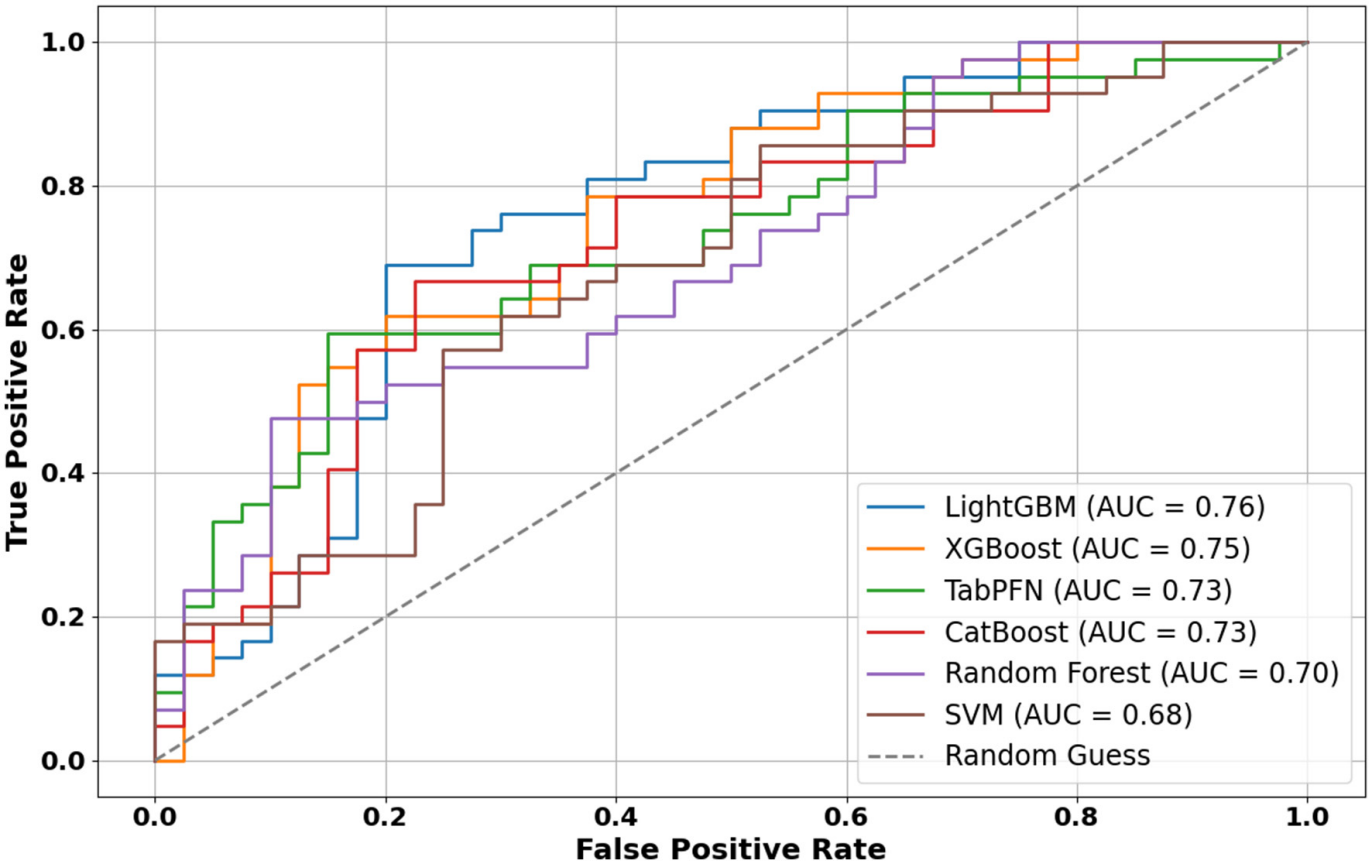
ROC-AUC curves for all models.

**Figure 3:**
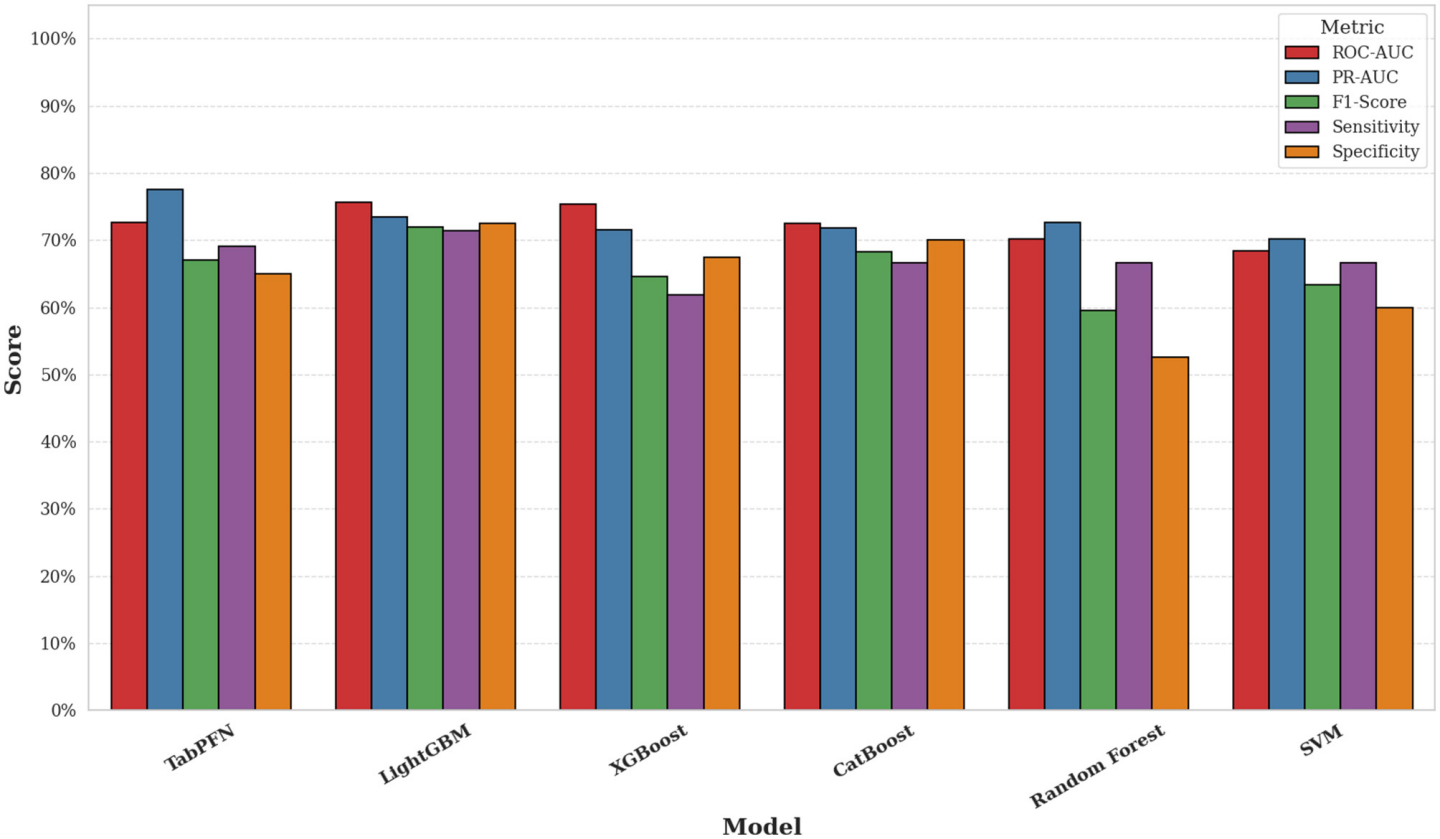
Comparison of classification metrics across tabular models.

The prediction task involved classifying infants into high vs. low-to-moderate negative emotionality groups using maternal mental health and demographic features. As shown in Figure 2, LightGBM achieved the highest ROC-AUC (0.76), followed closely by XGBoost (0.75), CatBoost (0.73), and TabPFN (0.73). Traditional classifiers such as Random Forest and SVM demonstrated lower ROC-AUC values of 0.70 and 0.68, respectively. Figure 3 shows a comparison of all performance metrics across all models. In terms of PR-AUC performance, TabPFN ranked highest with a PR-AUC of 0.78, followed by LightGBM and Random Forest (0.73 each), CatBoost and XGBoost (0.72 each), and SVM (0.70). Evaluation of F1-scores revealed that LightGBM outperformed other models (0.72), with CatBoost (0.69), TabPFN (0.67), and XGBoost (0.65) trailing closely, while SVM (0.63) and Random Forest (0.60) yielded the lowest scores. Sensitivity scores were highest for LightGBM (0.71), followed by TabPFN (0.69), and a cluster of models including CatBoost, Random Forest, and SVM (0.67 each); XGBoost showed the lowest sensitivity (0.62). Regarding specificity, LightGBM again led (0.73), followed by CatBoost (0.70), XGBoost (0.68), and TabPFN (0.65). The results of the repeated five-fold cross-validation are summarized in Table B1 of Appendix B.

### 3.3 Feature importance analysis

**Figure 4:**
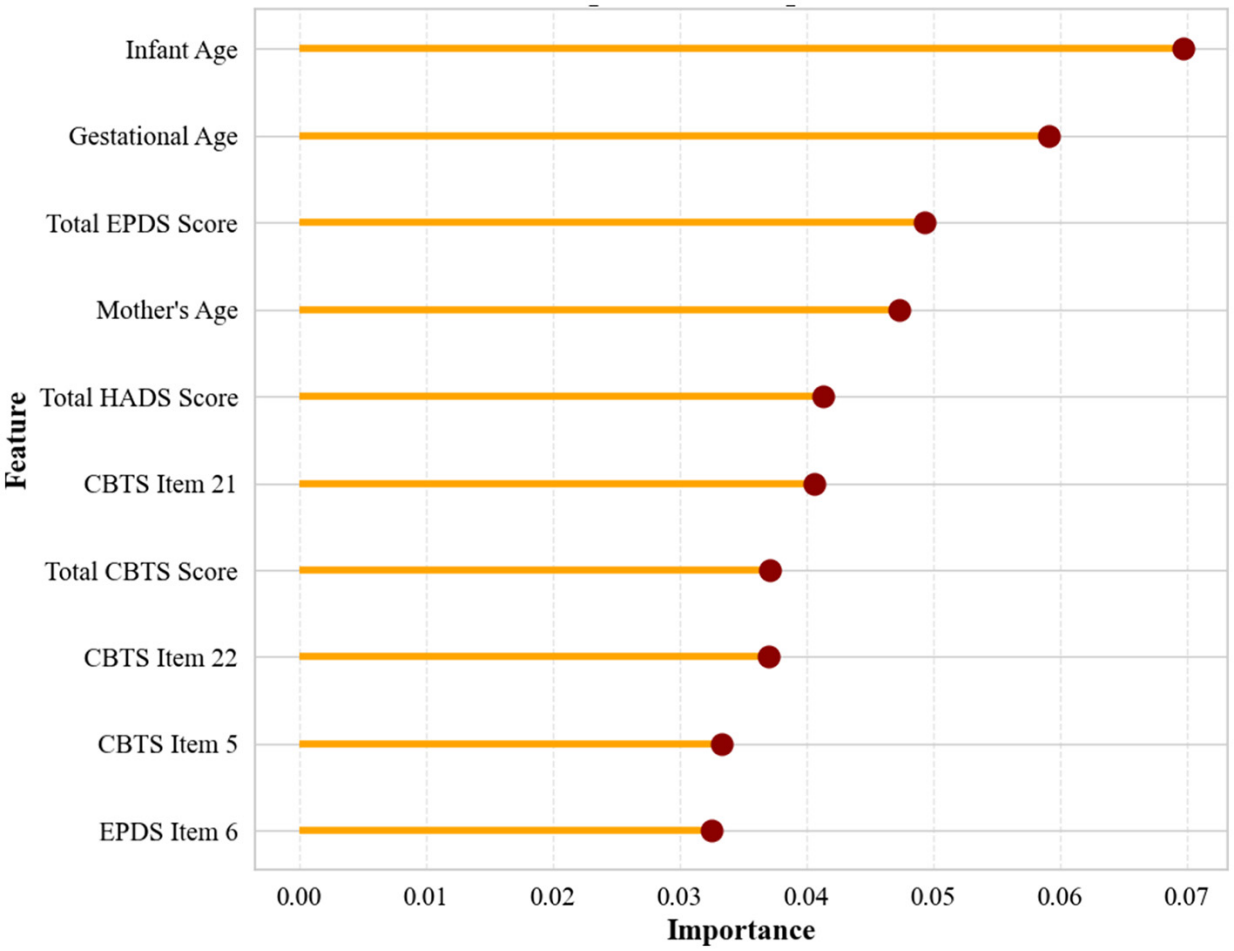
Top predictive features for infant negative emotionality.

Figure 4 presents the most important predictors of infant negative emotionality identified by our models. Gestational age emerged as the most important predictor, followed by the total EPDS score, maternal age, total HADS score, and total CBTS score. Infant age was also among the key features, along with individual items from the CBTS (Items 22, 21, and 5), EPDS (Items 6 and 3), and HADS (Item 7) scales.

## 4 Discussion

### 4.1 Main findings

This study investigated whether MMH measures, specifically depression, anxiety, and birth-related trauma, could predict infant negative emotionality during the first year postpartum, by evaluating the performance of six tabular ML models in classifying infants into high vs. low-to-moderate negative emotionality groups.

The findings show that ML models can moderately predict infant temperament based on MMH and demographic data, with performance varying across models and evaluation metrics. Ensemble gradient boosting models, LightGBM and XGBoost, consistently ranked among the top performers across ROC-AUC, F1-score, sensitivity, and specificity. LightGBM, in particular, achieved the highest ROC-AUC (0.76), F1-score (0.72), sensitivity (0.71), and specificity (0.73), highlighting its robust and balanced performance across key classification metrics. TabPFN, a state-of-the-art transformer-based model designed for tabular data, achieved the highest PR-AUC (0.78), indicating strong precision-recall performance, but lagged behind LightGBM and XGBoost in other metrics.

While TabPFN's PR-AUC performance is notable, its inability to outperform traditional ensemble models across all metrics may be attributed to several factors. First, TabPFN is trained on synthetic data from a large meta-distribution of tasks, and although it offers strong inductive biases for generalization, its zero-shot capabilities may not fully exploit the unique patterns present in small, domain-specific datasets such as ours. Additionally, the MMH features used, comprising structured questionnaire items and demographic variables, may be more effectively captured by tree-based models that inherently handle mixed data types, non-linearity, and feature interactions. In contrast, gradient boosting models like LightGBM and XGBoost are well-suited for structured tabular data. Their iterative boosting frameworks enable the capture of subtle feature contributions and non-linear relationships.

Overall, these results support the feasibility of using MMH indicators along with demographic data to identify infants at elevated risk of negative emotionality. While TabPFN shows promise, gradient boosting models remain more reliable for this prediction task given the current data structure and sample size.

### 4.2 Predictors of infant negative emotionality

Infant age emerged as the strongest predictor of negative emotionality. Previous studies have shown that as infants mature from 3 to 12 months, they gradually exhibit fewer signs of irritability and distress (23, 24). This is largely attributed to the development of self-soothing abilities and enhanced attentional control. These findings underline that infant age robustly shapes the expression and structure of temperament during infancy, reinforcing its significance as a predictive feature in our model.

Interestingly, gestational age at birth appeared as the second strongest predictor of negative emotionality during infancy. This finding aligns with prior literature indicating that preterm birth (birth before 37 weeks gestation) is associated with higher levels of negative emotional reactivity or fussiness in infancy (25). Moreover, studies involving both very preterm infants (born before 32 weeks of gestation) and moderate-to-late preterm infants (born between 32 and 36 weeks of gestation) indicate increased emotional dysregulation and greater affective instability compared with full-term peers (born between 37 and 42 weeks of gestation) (26, 27). Additionally, a meta-analysis (28) confirms that lower gestational age consistently correlates with higher negative emotionality. Collectively, these findings confirm the relevance of gestational age as a prominent predictor in the modeling of infant temperament. Crucially, the association between prematurity and early emotional dysregulation may set the stage for later behavioral and mental health risks (29–31).

Postpartum depression, as measured by elevated EPDS scores, has also been identified as a key predictor of infant negative emotionality. Elevated maternal depressive symptoms have been consistently linked to higher levels of infant negative emotionality, likely due to both biological and environmental factors (32, 33). Additional evidence indicates that even subclinical maternal depressive symptoms are linked to more negative maternal perceptions of infant crying, which can reinforce infant negative reactivity (34). These findings validate the EPDS total score as a significant and clinically meaningful predictor in our model's prediction of infant temperament.

Maternal age was also identified as a significant predictor of infant negative emotionality. Younger maternal age has been associated with elevated levels of infant irritability and distress, potentially due to limited parenting experience and emotional resources. In contrast, older maternal age is linked to improved emotional regulation and caregiving stability, contributing to calmer infant temperaments and enhanced self-regulation (35).

Postpartum anxiety, as measured by the total HADS score, was also a significant predictor of infant negative emotionality. Prior research shows that maternal anxiety influences infant affect through both genetic susceptibility and altered caregiving behaviors, such as heightened vigilance and emotional unavailability, which can amplify infant distress and reactivity (8, 36, 37).

Maternal difficulty concentrating, a core symptom of birth-related posttraumatic stress (CBTS Item 21), was identified as an important feature in predicting infant negative. It has been linked to reduced attentiveness in interactions, which can increase infant irritability and emotional reactivity (38). Additionally, neurobehavioral studies have shown that maternal cognitive strain adversely affects responsive parenting, which in turn can hinder infant emotion regulation development and shape more negative temperament profiles (39, 40).

The remaining predictors: CBTS total score, CBTS Items 22 and 5, and EPDS Item 6 further underscore the influence of maternal trauma and depressive symptoms on infant temperament. These items capture maternal emotional overwhelm, flashbacks, and feelings of self-blame, which may impair maternal sensitivity and regulation during caregiving, potentially intensifying infant distress and reinforcing patterns of negative emotionality early in development (41).

Collectively, these findings highlight the complex interplay between maternal mental health symptoms, developmental factors, and caregiving dynamics in predicting infant negative emotionality. It is important to note that a high negative emotionality does not indicate a developmental disorder but reflects a specific temperamental style. Infants with higher scores may be more sensitive or reactive to environmental stimuli and transitions, requiring different caregiving strategies to support emotional regulation. Early recognition of these patterns allows caregivers to tailor interactions, promoting healthy emotional development through consistent routines, gentle soothing, and responsive caregiving.

### 4.3 Research and clinical implications

The findings of this study have several important implications for both research and clinical practice. First, the results emphasize the critical role of maternal mental health, particularly depressive, anxious, and birth trauma-related symptoms, in shaping infant temperament, specifically negative emotionality. The strong predictive value of maternal and infant age, alongside mental health indicators, underscores the need for early identification of at-risk mother–infant dyads.

From a research perspective, this study demonstrates the utility of tabular machine learning models, particularly gradient boosting algorithms, for predicting complex early behavioral outcomes using postpartum mental health assessments. This approach allows for the integration of heterogeneous data to model non-linear associations that traditional statistical methods may overlook. It also opens the door for predictive frameworks that can be adapted across different populations and clinical contexts. Importantly, the successful application of machine learning in this context supports its use in future research aiming to integrate multimodal data sources, such as genetic, physiological, and wearable data for a more comprehensive understanding of early emotional development. Additionally, these models may aid in identifying modifiable intervention targets and distinct infant subgroups who could benefit from different caregiving strategies or psychosocial support. Finally, the predictive pipeline developed in our study could be refined into real-time decision-support systems for use in maternal-child health research and digital health applications, facilitating earlier and more personalized preventive care tailored to the specific needs of mother–infant dyads.

Clinically, the ability to identify infants at higher risk for elevated negative emotionality could inform early intervention strategies within pediatric and maternal mental health services. Routine screening for maternal depression, anxiety, and birth-related trauma in postpartum care settings may enable clinicians to anticipate infant emotional regulation challenges and initiate preventive strategies. Incorporating these assessments into primary care or well-baby visits could allow for early referral to parent-infant psychotherapy, attachment-based interventions, or targeted parenting programs. Additionally, educating caregivers about temperament traits and offering strategies for managing infant distress, such as responsive soothing, emotion coaching, and structured routines, may buffer long-term emotional and behavioral risks. Personalized support for at-risk mother–infant dyads could ultimately enhance developmental outcomes and family well being. To enhance clinical interpretability, future iterations can include inherently transparent baselines such as elastic-net logistic regression, generalized additive models, and explainable boosting machines, as well as monotonic constraints within boosting models, enabling coefficient-based or shape-function explanations while quantifying any accuracy trade-offs.

### 4.4 Limitations

This study has several limitations that should be considered when interpreting the findings. First, its cross-sectional design limits the ability to infer causality. While we acknowledge the bidirectional nature of the relationship between MMH and infant temperament, our modeling framework treated maternal factors solely as predictors and infant temperament as a static outcome. This approach was necessitated by the cross-sectional design, which precludes examining temporal dynamics or reciprocal influences over time. Future research employing longitudinal data and advanced modeling frameworks—such as joint prediction models or probabilistic graphical models—could better capture these recursive feedback loops and clarify the directionality of effects. Second, all data were based solely on maternal self-report measures, which may introduce reporting biases and shared method variance. This reliance on a single informant for both predictor and outcome measures may inflate observed associations, as mothers experiencing higher psychological symptoms could perceive or report their infant's behaviors differently. Future studies incorporating multi-informant reports or objective behavioral assessments could mitigate this potential bias. Third, we did not explore ensemble approaches that combine predictions from multiple models (e.g., stacking, blending, or voting) to potentially improve accuracy and robustness. Our primary aim was to benchmark and compare the performance of individual tabular machine learning models. Future work could investigate ensemble strategies, which may leverage complementary strengths of different algorithms to enhance predictive performance. While tree-based models are generally robust to multicollinearity, SVMs—particularly those with linear kernels—can be affected by highly correlated predictors. Although we employed an RBF kernel, which is less sensitive to multicollinearity, this limitation should still be considered when interpreting results. Furthermore, although infant age emerged as a top predictor in our models, we did not stratify model training by age group due to the limited sample size. Our dataset included three infant age groups; dividing the total sample of 410 dyads across these groups would have substantially reduced the number of observations available for model training in each infancy group, risking overfitting and reduced generalizability. Future studies with larger datasets could examine age-stratified models to assess whether predictive performance and feature importance profiles differ across developmental stages within the first year. Finally, the models included a limited set of maternal psychological and demographic predictors. Important contextual factors—such as paternal mental health, caregiving dynamics, socioeconomic stressors, sleep patterns, and infant feeding—were not captured. Including multimodal data from diverse sources in future research would strengthen predictive accuracy and enhance the ecological validity of infant temperament modeling.

## 5 Conclusion

This study demonstrates the feasibility of using maternal mental health indicators and demographic variables to predict infant negative emotionality during the first year postpartum using tabular machine learning models. Among the six models tested, LightGBM and TabPFN showed the highest predictive performance across multiple evaluation metrics. Key predictors included gestational age, infant age, and maternal depression, anxiety, and birth-related trauma scores. These findings highlight the importance of integrating maternal mental health screening into postnatal care and underscore the potential of ML tools to support early identification of infants at risk for difficult temperament. By leveraging structured postpartum data, ML models can inform timely, targeted interventions to promote healthy infant development and enhance maternal-infant well being. Future work should expand on these findings using longitudinal and multimodal datasets to refine predictive accuracy and develop practical, scalable decision-support tools for clinical and community settings.

## Data Availability

The original contributions presented in the study are included in the article/Supplementary material, further inquiries can be directed to the corresponding author. The data used in this study are publicly available and can be accessed through the Zenodo repository at https://doi.org/10.5281/zenodo.5070945.
