# Supplementary material for: From mother to infant: predicting infant temperament using maternal mental health measures and tabular machine learning models
Source: Front Public Health. 2025 Sep 18;13:1659987. doi: 10.3389/fpubh.2025.1659987 (PMC12490419; doi:10.3389/fpubh.2025.1659987)
Supplement: Supplementary file 1 [file Data_Sheet_1.docx]

**Appendix A: Detailed List of Variables**

| **Variable name** | **Variable description** | **Additional information** |
| --- | --- | --- |
|  | | |
| **A.1 Demographic and Maternal Variables** | | |
| Mother's age | Item determining participant's age in years | min = 19, max = 47 |
| Mother’s education | Item determining participant's educational level | 1 = no education; 2 = compulsory school; 3 = post-compulsory education; 4 = university of Applied Science or University Technology Degree; 5 = university. |
| Gestational age | Item determining the infant weeks of gestation | min = 26, max = 43 |
| Pregnancy type | Item determining the type of pregnancy | 1 = single pregnancy; 2 = twin pregnancy (only 1st baby info is used in the analysis) |
| Infant age | Item determining the age category of the infant | 1 = ≥3 months to <6 months; 2 = ≥6 months to <9 months; 3 = ≥9 months to <12 months |
| Infant sex | Item determining the sex of the infant | 1 = girl; 2 = boy |
|  | | |
| **A.2 Edinburgh Postnatal Depression Scale** | | |
| EPDS_1 | Item 1 of the Edinburgh Postnatal Depression Scale | Q: I have been able to laugh and see the funny side of things. |
| EPDS_2 | Item 2 of the Edinburgh Postnatal Depression Scale | Q: I have felt confident and joyful when thinking about the future. |
| EPDS_3 | Item 3 of the Edinburgh Postnatal Depression Scale | Q: I have blamed myself unnecessarily when things went wrong. |
| EPDS_4 | Item 4 of the Edinburgh Postnatal Depression Scale | Q: I have felt anxious or worried for no good reason. |
| EPDS_5 | Item 5 of the Edinburgh Postnatal Depression Scale | Q: I have felt scared or panicked for no real reason. |
| EPDS_6 | Item 6 of the Edinburgh Postnatal Depression Scale | Q: I have felt overwhelmed by events. |
| EPDS_7 | Item 7 of the Edinburgh Postnatal Depression Scale | Q: I have felt so unhappy that I had trouble sleeping. |
| EPDS_8 | Item 8 of the Edinburgh Postnatal Depression Scale | Q: I have felt sad or not very happy. |
| EPDS_9 | Item 9 of the Edinburgh Postnatal Depression Scale | Q: I have felt so unhappy that I have cried. |
| EPDS_10 | Item 10 of the Edinburgh Postnatal Depression Scale | Q: I have had thoughts of harming myself. |
|  | | |
| **A.3 Hospital Anxiety and Depression Scale (anxiety subscale)** | | |
| HADS_1 | Item of the Hospital Anxiety and Depression Scale (anxiety subscale) | Q: I feel tense or nervous. |
| HADS_3 | Item of the Hospital Anxiety and Depression Scale (anxiety subscale) | Q: I have a feeling of fear as if something terrible is going to happen. |
| HADS_5 | Item of the Hospital Anxiety and Depression Scale (anxiety subscale) | Q: I worry a lot. |
| HADS_7 | Item of the Hospital Anxiety and Depression Scale (anxiety subscale) | Q: I can sit quietly and feel relaxed. |
| HADS_9 | Item of the Hospital Anxiety and Depression Scale (anxiety subscale) | Q: I experience feelings of fear and have a knot in my stomach. |
| HADS_11 | Item of the Hospital Anxiety and Depression Scale (anxiety subscale) | Q: I feel restless and can’t seem to stay still. |
| HADS_13 | Item of the Hospital Anxiety and Depression Scale (anxiety subscale) | Q: I have sudden feelings of panic. |
|  | | |
| **A.4 Maternal City Birth Trauma Scale** | | |
| CBTS_M_3 | Item 3 of the maternal City Birth Trauma Scale | Q: Repeated and involuntary memories of the birth (or parts of the birth) that you cannot control. |
| CBTS_M_4 | Item 4 of the maternal City Birth Trauma Scale | Q: Bad dreams or nightmares about the birth or related to the birth. |
| CBTS_M_5 | Item 5 of the maternal City Birth Trauma Scale | Q: Flashbacks of the birth and/or reliving the experience. |
| CBTS_M_6 | Item 6 of the maternal City Birth Trauma Scale | Q: Feeling distressed when something reminds you of the birth. |
| CBTS_M_7 | Item 7 of the maternal City Birth Trauma Scale | Q: Feeling tense or anxious when something reminds you of the birth. |
| CBTS_M_8 | Item 8 of the maternal City Birth Trauma Scale | Q: Trying to avoid thinking about the birth. |
| CBTS_M_9 | Item 9 of the maternal City Birth Trauma Scale | Q: Trying to avoid things that remind you of the birth (e.g., people, places, TV shows, etc.). |
| CBTS_M_10 | Item 10 of the maternal City Birth Trauma Scale | Q: Unable to remember details of the birth. |
| CBTS_M_11 | Item 11 of the maternal City Birth Trauma Scale | Q: Blaming yourself or others for what happened during the birth. |
| CBTS_M_12 | Item 12 of the maternal City Birth Trauma Scale | Q: Having intense negative emotions about the birth (e.g., fear, anger, shame). |
| CBTS_M_13 | Item 13 of the maternal City Birth Trauma Scale | Q: Having negative beliefs about yourself or fearing that something terrible will happen. |
| CBTS_M_14 | Item 14 of the maternal City Birth Trauma Scale | Q: Losing interest in activities that were previously important to you. |
| CBTS_M_15 | Item 15 of the maternal City Birth Trauma Scale | Q: Feeling detached from others. |
| CBTS_M_16 | Item 16 of the maternal City Birth Trauma Scale | Q: Unable to feel positive emotions (e.g., joy, excitement). |
| CBTS_M_17 | Item 17 of the maternal City Birth Trauma Scale | Q: Feeling irritable or aggressive. |
| CBTS_M_18 | Item 18 of the maternal City Birth Trauma Scale | Q: Having self-destructive feelings or engaging in reckless behavior. |
| CBTS_M_19 | Item 19 of the maternal City Birth Trauma Scale | Q: Feeling tense and on edge. |
| CBTS_M_20 | Item 20 of the maternal City Birth Trauma Scale | Q: Feeling restless or easily startled. |
| CBTS_M_21 | Item 21 of the maternal City Birth Trauma Scale | Q: Having difficulty concentrating. |
| CBTS_M_22 | Item 22 of the maternal City Birth Trauma Scale | Q: Having trouble sleeping due to reasons unrelated to the baby’s sleep pattern. |
|  | | |
| **A.5 Infant Behavior Questionnaire-Revised Negative Emotionality (IBQ-NEG) items.** | | |

| IBQ_R_VSF_3_bb1 | Item 3 of the the Very Short Form of the Infant Behavior Questionnaire-Revised (Negative Emotionality dimension) | When your baby was tired, how often did he/she show distress? |
| --- | --- | --- |
| IBQ_R_VSF_4_bb1 | Item 4 of the the Very Short Form of the Infant Behavior Questionnaire-Revised (Negative Emotionality dimension) | When you introduced your baby to an unfamiliar adult, how often did he/she cling to a parent? |
| IBQ_R_VSF_9_bb1 | Item 9 of the the Very Short Form of the Infant Behavior Questionnaire-Revised (Negative Emotionality dimension) | When it was bedtime or naptime and your baby did not want to go, how often did he/she whine or sob? |
| IBQ_R_VSF_10_bb1 | Item 10 of the the Very Short Form of the Infant Behavior Questionnaire-Revised (Negative Emotionality dimension) | After sleep, how often did your baby cry if no one arrived quickly? |
| IBQ_R_VSF_16_bb1 | Item 16 of the the Very Short Form of the Infant Behavior Questionnaire-Revised (Negative Emotionality dimension) | How often did your baby seem angry (crying and fussing) when you put him/her to bed? |
| IBQ_R_VSF_17_bb1 | Item 17 of the the Very Short Form of the Infant Behavior Questionnaire-Revised (Negative Emotionality dimension) | How often in the past week was your baby startled by a sudden change in body position (e.g., when moved suddenly)? |
| IBQ_R_VSF_28_bb1 | Item 28 of the the Very Short Form of the Infant Behavior Questionnaire-Revised (Negative Emotionality dimension) | When you introduced your baby to an unfamiliar adult, how often did he/she refuse to go to that person? |
| IBQ_R_VSF_29_bb1 | Item 29 of the the Very Short Form of the Infant Behavior Questionnaire-Revised (Negative Emotionality dimension) | When you were busy with another activity and your baby could not get your attention, how often did he/she cry? |
| IBQ_R_VSF_32_bb1 | Item 32 of the the Very Short Form of the Infant Behavior Questionnaire-Revised (Negative Emotionality dimension) | When your baby wanted something, how often did he/she get upset when unable to get what he/she wanted? |
| IBQ_R_VSF_33_bb1 | Item 33 of the the Very Short Form of the Infant Behavior Questionnaire-Revised (Negative Emotionality dimension) | When in the presence of multiple unfamiliar adults, how often did your baby cling to a parent? |

**Appendix B: Cross-Validation Performance Variability Across Models**

**Appendix Table B1.** Cross-validation mean scores (± standard deviation) for all models across evaluation metrics. *TabPFN results are reported as single estimates without variability measures due to its zero-shot architecture.*

| **Model** | **ROC-AUC (%)** | **PR-AUC (%)** | **F1-Score (%)** | **Sensitivity (%)** | **Specificity (%)** |
| --- | --- | --- | --- | --- | --- |
| **TabPFN** | 72.5 | 77.5 | 67.0 | 69.0 | 65.0 |
| **LightGBM** | 75.5 ± 0.9 | 73.0 ± 1.4 | 72.0 ± 1.1 | 71.5 ± 0.8 | 72.5 ± 1.7 |
| **XGBoost** | 75.0 ± 1.2 | 71.5 ± 1.0 | 64.5 ± 1.9 | 62.0 ± 1.6 | 67.0 ± 1.3 |
| **CatBoost** | 72.5 ± 1.4 | 71.5 ± 1.5 | 68.5 ± 0.9 | 66.5 ± 1.2 | 70.0 ± 1.8 |
| **Random Forest** | 70.0 ± 1.6 | 72.5 ± 0.7 | 59.5 ± 1.8 | 66.5 ± 1.1 | 52.5 ± 1.9 |
| **SVM** | 68.5 ± 1.3 | 70.0 ± 1.6 | 63.5 ± 0.8 | 66.5 ± 1.7 | 60.0 ± 1.2 |
